# Anti-tumor pharmacology of natural products targeting mitosis

**DOI:** 10.20892/j.issn.2095-3941.2022.0006

**Published:** 2022-06-15

**Authors:** Manru Huang, Caiyan Liu, Yingying Shao, Shiyue Zhou, Gaoyong Hu, Shuangshuang Yin, Weiling Pu, Haiyang Yu

**Affiliations:** 1 Key Laboratory of Pharmacology of Traditional Chinese Medical Formulae, Ministry of Education, Tianjin University of Traditional Chinese Medicine, Tianjin 301617, China; 2 State Key Laboratory of Component-based Chinese Medicine, Tianjin University of Traditional Chinese Medicine, Tianjin 301617, China

**Keywords:** Tumor, mitosis, natural products, mechanism, pharmacology

## Abstract

Cancer has been an insurmountable problem in the history of medical science. The uncontrollable proliferation of cancer cells is one of cancer’s main characteristics, which is closely associated with abnormal mitosis. Targeting mitosis is an effective method for cancer treatment. This review summarizes several natural products with anti-tumor effects related to mitosis, focusing on targeting microtubulin, inducing DNA damage, and modulating mitosis-associated kinases. Furthermore, the main disadvantages of several typical compounds, including drug resistance, toxicity to non-tumor tissues, and poor aqueous solubility and pharmacokinetic properties, are also discussed, together with strategies to address them. Improved understanding of cancer cell mitosis and natural products may pave the way to drug development for the treatment of cancer.

## Introduction

According to data reported by International Agency for Research on Cancer, approximately 19.3 million new cancer cases and 10 million cancer deaths occurred worldwide in 2020^1^. Although effective drugs for cancer treatment are continually being developed, cancer mortality is increasing each year^2^. Compared with normal cells, cancer cells have characteristics of uncontrollable proliferation, evasion of growth inhibitors, invasion and metastasis, and evasion of immune surveillance, thus making cancer difficult to cure and resulting in poor prognosis^3^. The uncontrollable proliferation of cancer cells, a major characteristic of cancer, is closely associated with abnormal mitosis. The mitosis of cancer cells is often unregulated, and cell division is accelerated by cancer cell evasion of growth inhibitory factors^4^. Targeting proliferation or mitosis are effective methods for cancer treatment. Many natural products exert favorable anti-tumor effects through regulating mitosis. The classical anti-mitotic drugs derived from natural products, including periwinkle alkaloids or paclitaxel-like drugs and their derivatives, have a wide range of clinical uses^5^.

Microtubules are involved in segregation during mitosis in eukaryotic cells. Microtubules are regulated primarily by polymerization kinetics, and their polymerization occurs through a nucleation-extension mechanism^6^. Targeting microtubules is currently used to prevent tumor cell mitosis and retard tumor development in clinical therapy^7^. DNA damage is directly associated with normal mitotic processes. If DNA damage is not repaired before mitosis occurs, the probability of chromosomal mutations is greatly increased, and the damaged DNA can enter daughter cells through faulty mitosis, and eventually induce cellular lesions or death^8,9^. Thus, promoting DNA damage in cancer cells and inhibiting repair can decrease cancer cell proliferation or promote cancer cell death. The growth of cancer cells is faster than that of normal cells, because cancer cells can avoid the inhibition of growth factors and unscheduled re-entry into the cell cycle^10^. Beyond microtubules and DNA damage, mitosis-associated kinases are another important aspect of mitosis that can be targeted. These kinases include cyclin-dependent kinases (CDKs) and aurora kinases (AURKs). Numerous CDKs regulate cell mitosis through binding cyclin proteins. The first CDK inhibitor to enter clinical studies was flavopiridol, derived from the native Indian plant rohitukine^11^. AURKs are major serine/threonine kinases in mitosis, which are involved in several events such as centrosome maturation and segregation, spindle assembly and maintenance, chromosome segregation, and cytoplasmic segregation. Studies have shown that inhibition of AURK expression or activity inhibits the proliferation, migration, and invasion of cancer cells^12^. Studies are identifying an increasing number of natural products that downregulate AURKs, and the modification of AURKA inhibitors may be a promising research direction.

This review summarizes several natural products with anti-tumor effects related to mitosis, focusing on targeting microtubulin, DNA damage, and mitosis-associated kinases. We also briefly summarize the mechanisms of mitosis targeting by these natural products in **Table 1**, and clinically developed antitumor drugs targeting mitosis derived from natural products in **Table 2**. Improved understanding of cancer cell mitosis and natural products may pave the way to further drug development for the treatment of cancer.

**Table 1:** Summary of anti-tumor natural products targeting mitosis

Herbal medicine	Source	Chemical structure	Target	Mechanism	Indications	References
**Paclitaxel (Taxol)**	*Taxus brevifolia Nutt.*	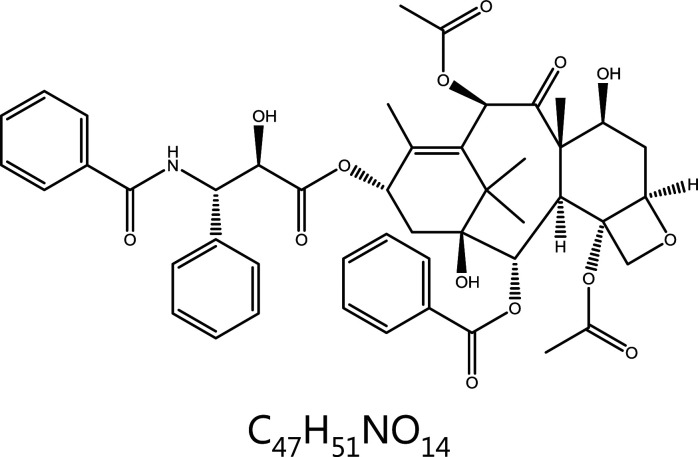	Microtubules	Binds the paclitaxel site and causes microtubule polymerization	Breast cancer, ovarian cancer, head and neck cancer, lung cancer, etc.	^18–22^
**Vincristine**	*Catharanthus roseus* (L.) G. Don	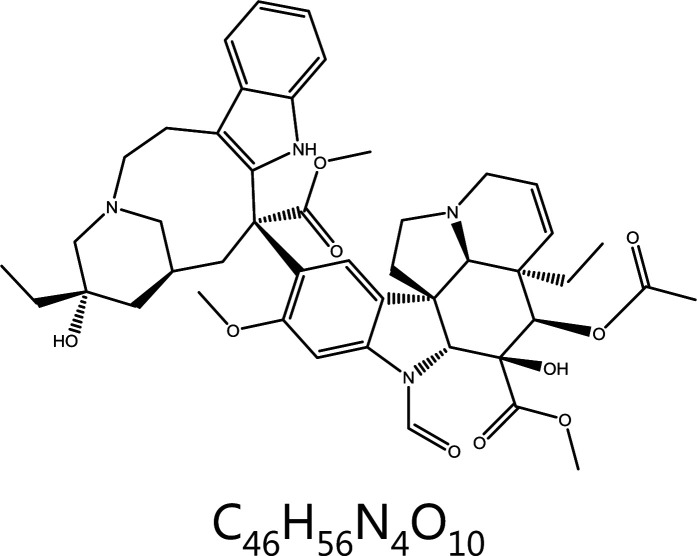	Microtubules	Binds the vincristine site and depolymerizes microtubules	Acute lymphoblastic leukemia, Hodgkin’s disease, reticulocytic sarcoma, breast cancer, etc.	^23,25,29^
**Colchicine**	*Colchicum autumnale* L.	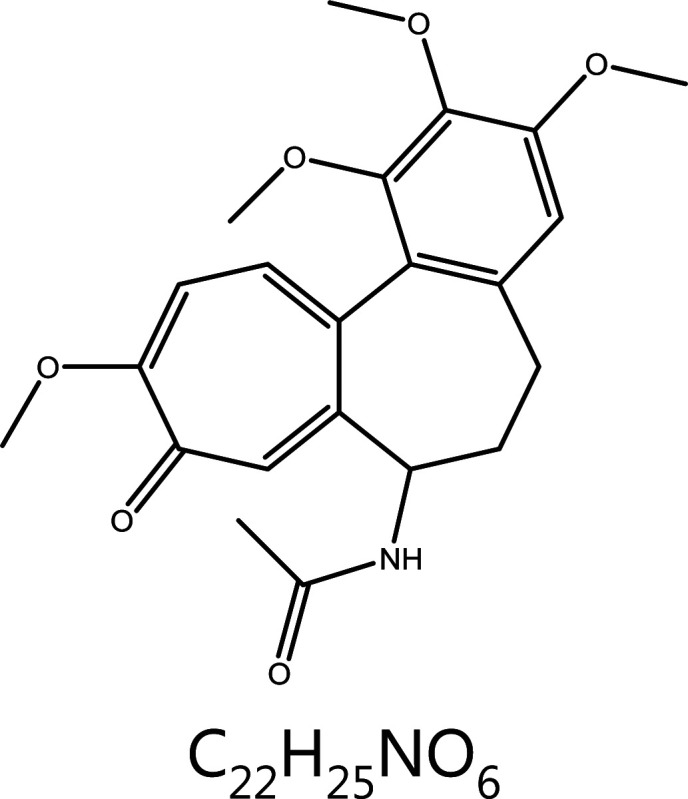	Microtubules	Binds colchicine sites and depolymerizes microtubules	Gout	^33,34^
**Podophyllotoxin**	*Sinopodophyllumhexandrum* (Royle) Ying	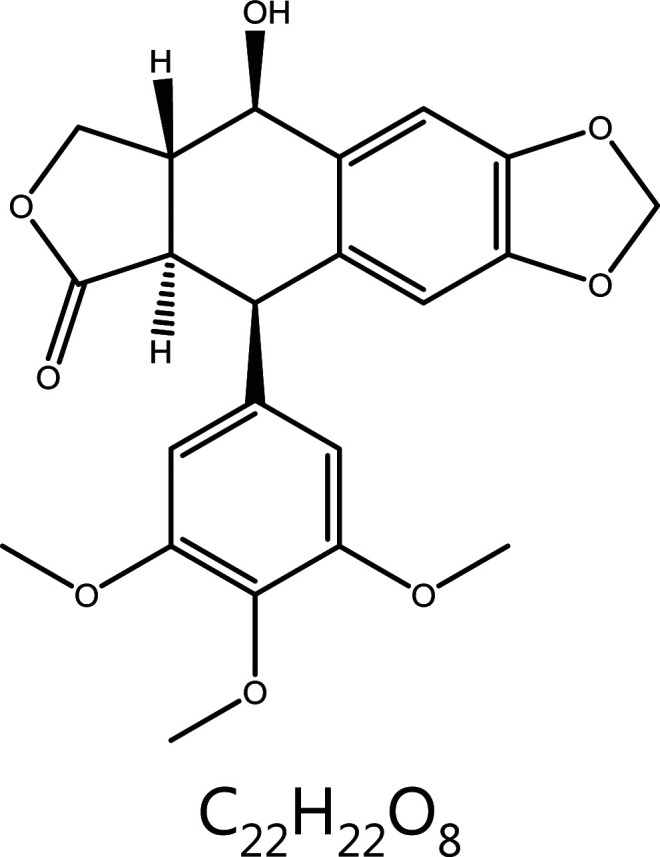	Microtubules	Binds colchicine sites and depolymerizes microtubules	Condyloma acuminatum, acute granulocytic leukemia, chronic eosinophilic leukemia, small cell lung cancer, etc.	^37–40^
**Aplyronine A**	*Aplysia kurodai*	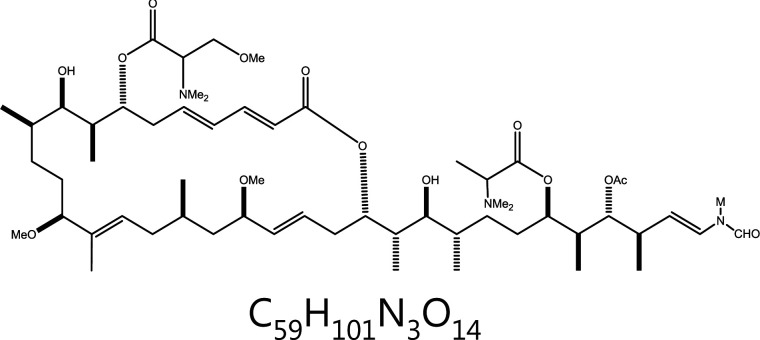	Microtubules	Microtubule depolymerization	–	^41,42,45,46^
**Pironetin**	Streptomyces	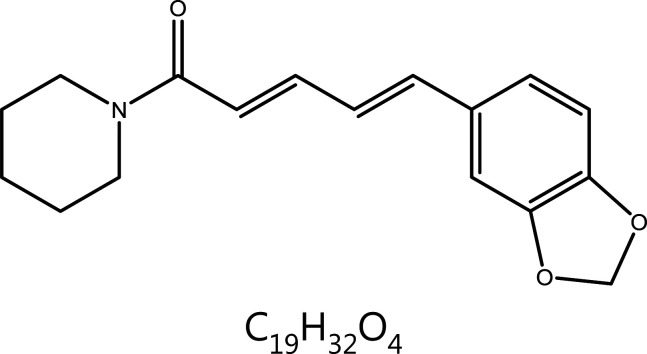	Microtubules	Binds alpha-microtubule protein and depolymerizes microtubules	–	^48,49^
**Camptothecin**	*Camptotheca acuminata* Decne.	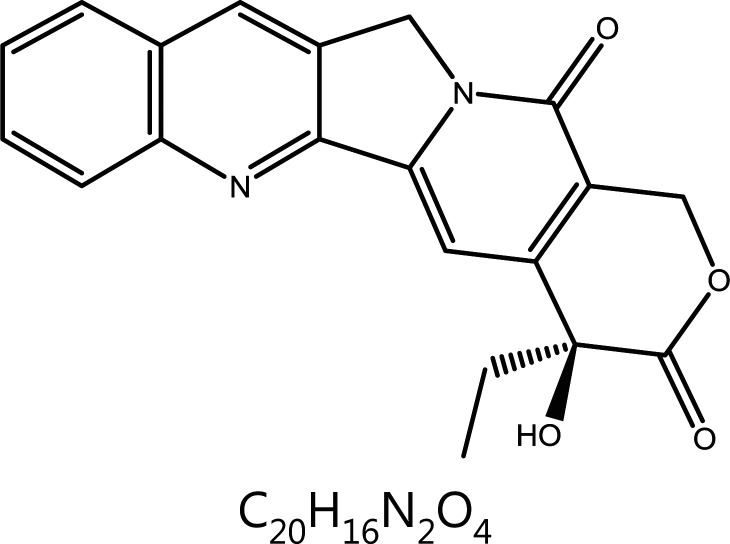	DNA damage	Topoisomerase I↓	Stomach cancer, esophageal cancer, colorectal cancer, primary liver cancer, lung cancer, etc.	^54,61^
**Piperine**	Piper Nigrum L.	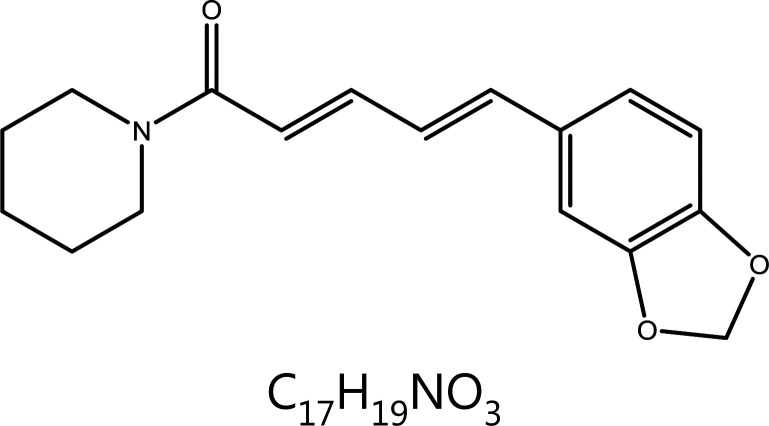	DNA damage	ROS↑, tetraspiral DNA molecule↑, DNA polymerase β↓	Convulsions, epilepsy, etc.	^65–69,71–73^
**Sulforaphane**	Brassica oleracea L.var.italic Planch.	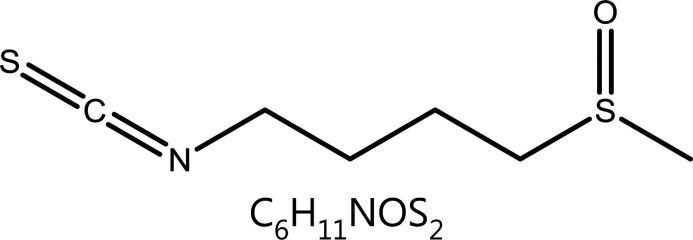	DNA damage	ROS↑, HDACs↓, γH2AX↑	Chronic obstructive pulmonary disease	^74–80^
**Resveratrol**	*Veratrum grandiflorum* Loes.	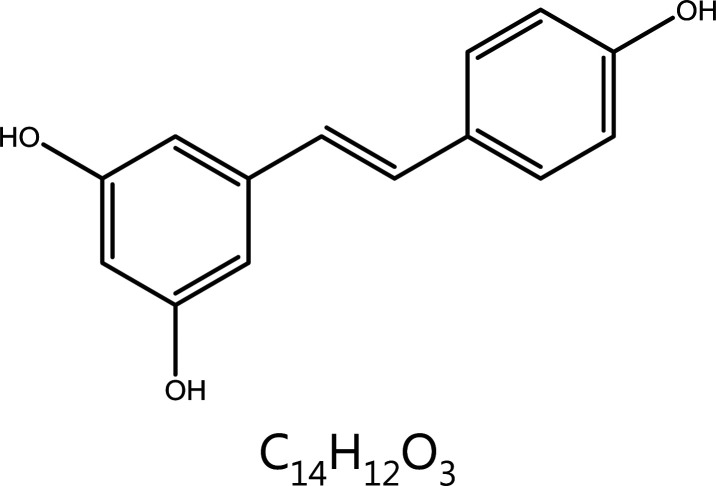	Cell cycle block	DDR↑, S phase block, apoptosis↑	Atherosclerosis, coronary artery disease, ischemic heart disease, hyperlipidemia, breast cancer, etc.	^81–84,86–89^
**Licochalcone A**	Glycyrrhiza uralensis Fisch.	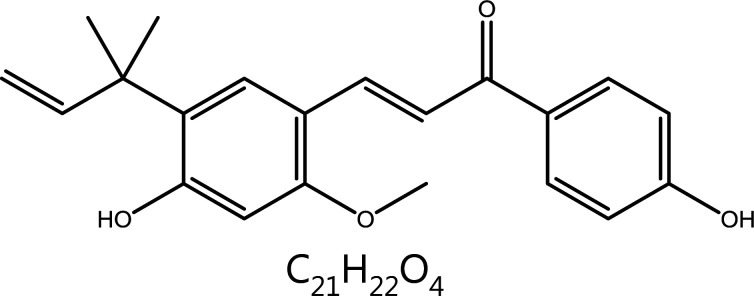	Cell cycle block	Cyclin B1↓, CDK1↓, G2/M phase block	–	^91^
**Hinokitiol**	*Chamaecyparisobtusa var. formosana* (Hayata) Rehd.	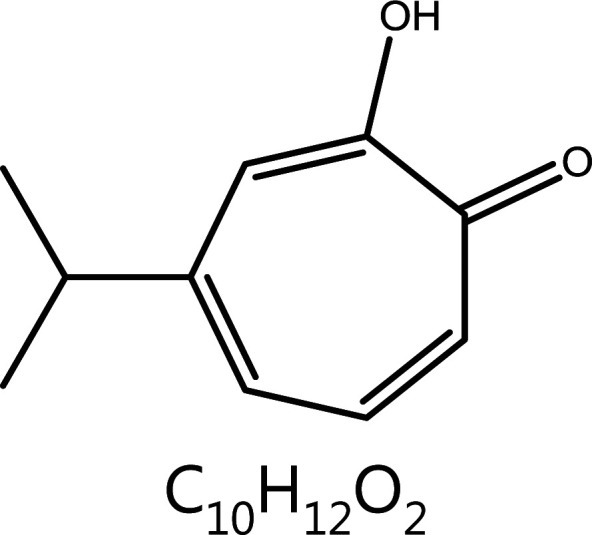	Cell cycle block	P53↑, D1-CDK4↓, G0/G1 phase block CDK7↓, Cyclin D1↓, Cyclin A2↓, S phase block	Inhibition of Staphylococcus aureus	^92,93^
**Cryptotanshinone**	*Salvia miltiorrhizaBge.*	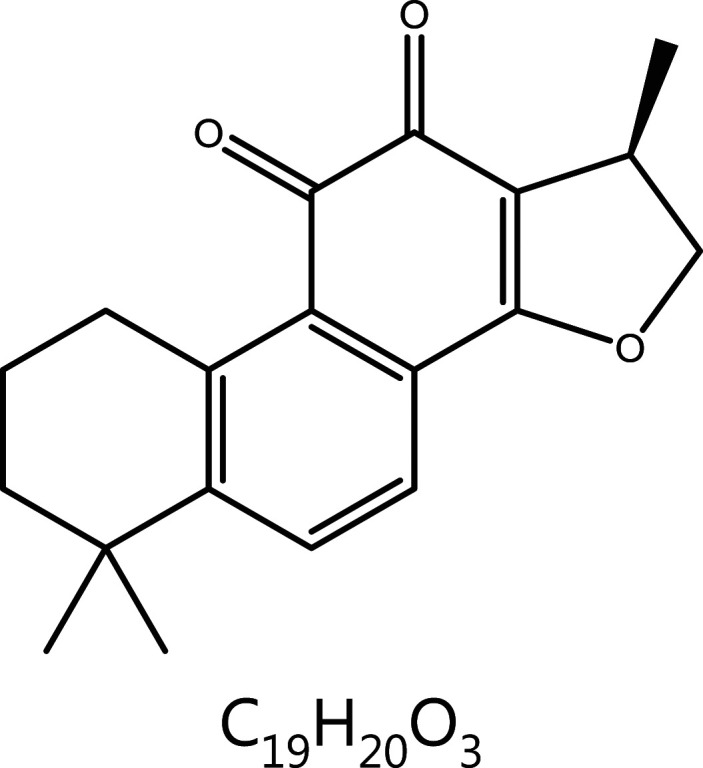	Cell cycle block	Cyclin A/D↓, CDK2/4↓, G0/G1 phase block	Coronary heart disease, angina pectoris, etc.	^94^
**Genistein**	*Euchresta japonica Hook. f. ex Regel *	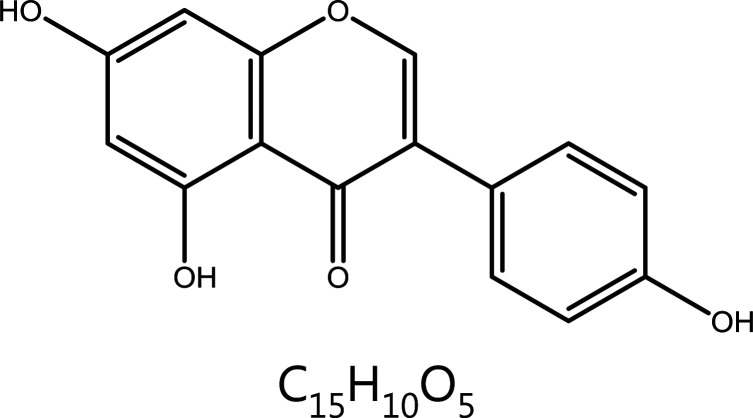	Cell cycle block	Cyclin A/B↓, CDK2/CDC2↓, G2/M phase block p15↑, CDK↓, p21↑, Cyclin D1↓	–	^95–99^
**Berberine**	*Coptis chinensis *Franch.	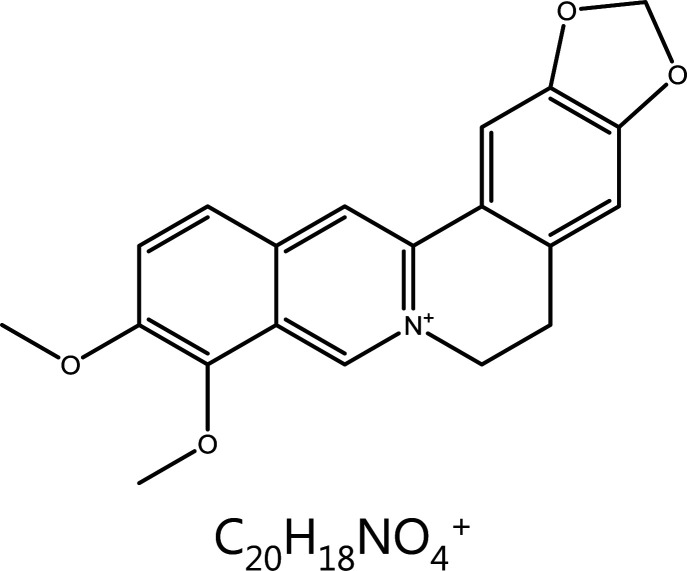	Cell cycle block	Cyclin D1/D2/E↓, CDK2/4/6↓, G1 phase block	Gastroenteritis, bacterial dysentery and other intestinal infections, conjunctivitis, suppurative otitis media, etc.	^100,101^
**Ascleposide**	*Saussurea lappa*	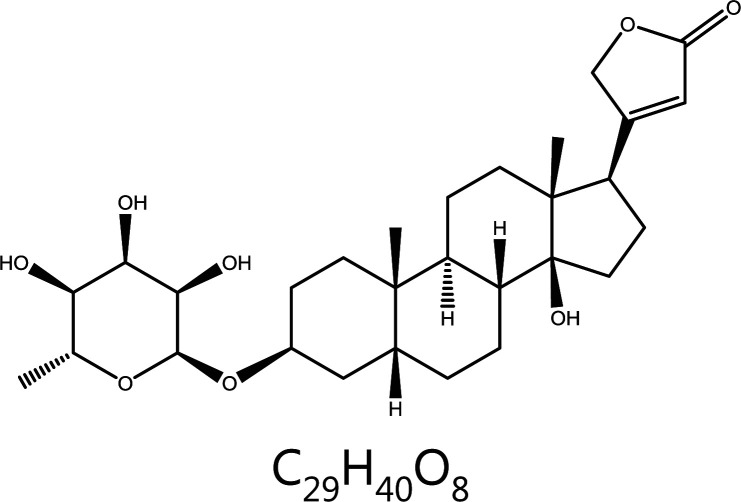	Cell cycle block	Cyclin D1/A↓, CDK4↓, p21/27↓, G2/M phase block	–	^102–104^
**Garcinol**	*Garcinia*	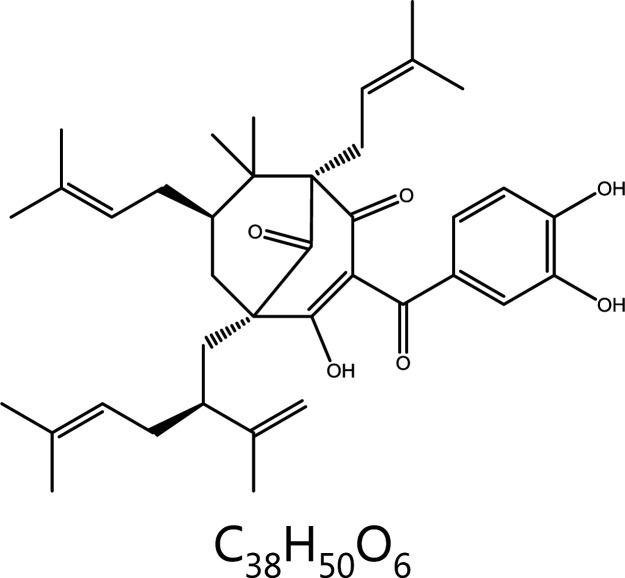	Cell cycle block	Cyclin D1/D3↓, CDK2/4↓ Cyclin E↑, CDK6↑, p21/27↑, G1 phase block	–	^108^
**Curcumin**	*Curcuma longa* L.	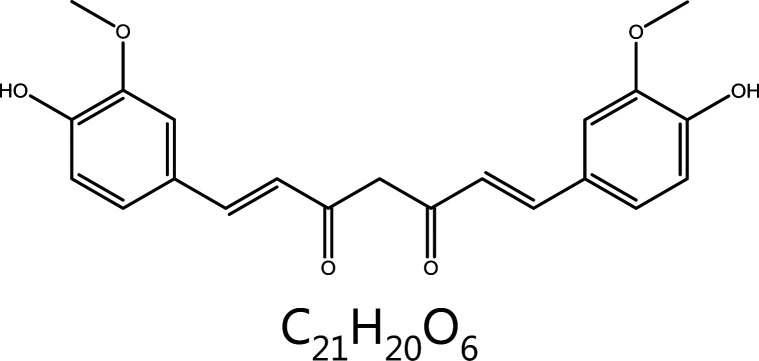	Aurora kinase	AURKA↓, Histone H3↓, monopolar spindle↑	Liver cancer, liver fibrosis, hyperlipidemia, Alzheimer’s disease, atherosclerosis, etc.	^124,125^
**Tanshinone I**	*Salvia miltiorrhizaBge.*	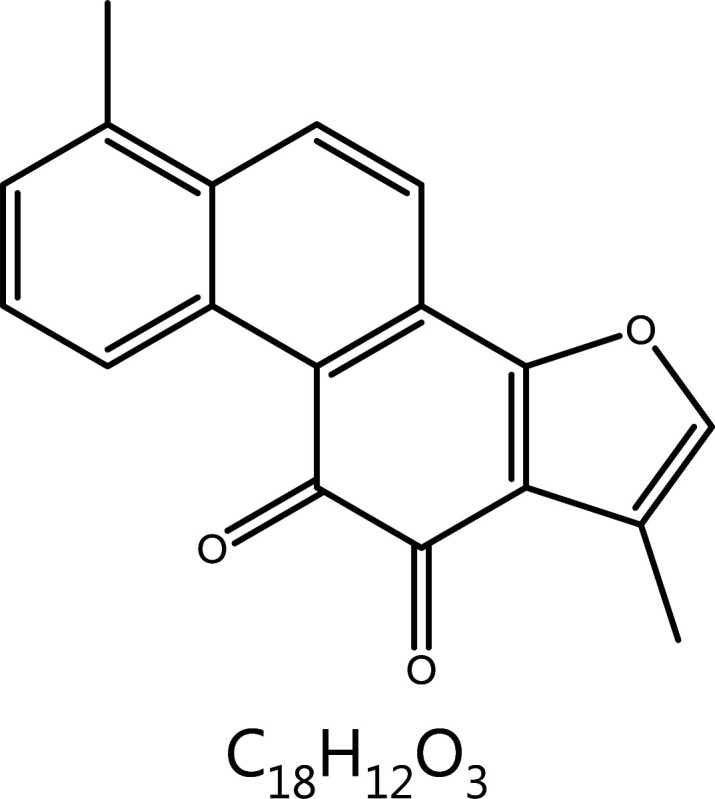	Aurora kinase	AURKA↓	Angina pectoris, coronary artery disease, etc.	^126–128^
**Gossypin**	*Hibiscus vitifolius*	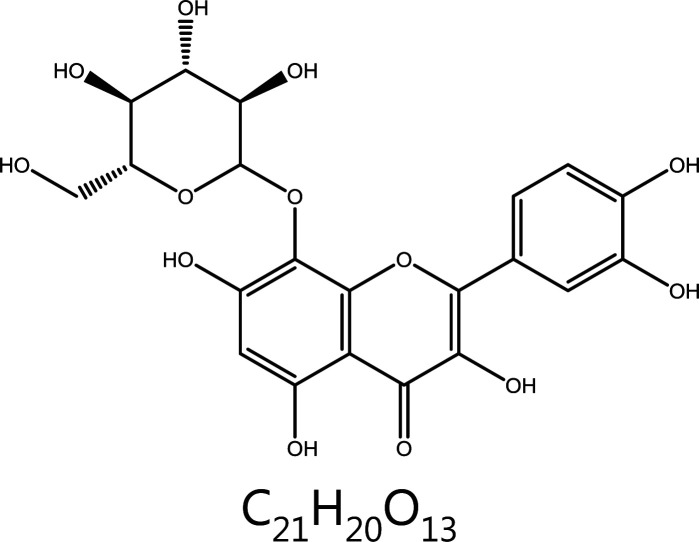	Aurora kinase	Binding ATP pocket of AURKA, AURKA↓	–	^129–131^

↑: promotes, ↓: inhibits.

**Table 2:** Summary of clinical drugs derived from natural products that target mitosis

Medicine	Source	Mechanisms	Indications	Approval status	References
**Taxol**	Paclitaxel	Maintains microtubule protein stability by inhibiting microtubule depolymerization and further inhibits cell mitosis	Ovarian cancer, breast cancer, lung cancer, head and neck cancer, esophageal cancer, germ cell tumors, endometrial cancer, lymphoma, bladder cancer, etc.	First marketed in 1992	^24–26^
**Abraxane**			Metastatic breast cancer that has failed combination chemotherapy or that has recurred within 6 months after adjuvant chemotherapy	First marketed in 2005	^27,28^
**Cynviloq**			Breast cancer, non-small cell lung cancer, etc.	First marketed in 2007	–
**Paclical**			Lung cancer, breast cancer, ovarian cancer, etc.	First marketed in 2015	^29^
**Liporaxel**			Breast cancer, stomach cancer, etc.	First marketed in 2016	^32^
**Oraxol**			Metastatic breast cancer, etc.	In phase III clinical trials	^33,34^
**Taxotere**			Breast cancer and non-small cell lung cancer, etc.	First marketed in 1996	^35,36^
**Jevtana**			Prostate cancer, etc.	First marketed in 2010	–
**Larotaxel**			Breast cancer, etc.	In phase III clinical trials	–
**Milataxel**			Breast cancer, colorectal cancer, etc.	In phase II clinical trials	–
**Ortataxel**			Breast cancer, etc.	In phase II clinical trials	–
**Vinblastine** **Sulfate**	Vinblastine	Prevents the formation of spindle microtubules by inhibiting microtubule protein polymerization, causing mitosis to stop at mid-phase	Malignant lymphoma, testicular tumor, choriocarcinoma, breast cancer, etc.	First marketed in 1958	^45,46^
**Vincristine** **Sulfate**	Vincristine		Leukemia, lymphoma, etc.	First marketed in 1962	^45,46^
**Vindesine** **Sulfate**	Vinblastine		Non-small cell lung cancer, small cell lung cancer, malignant lymphoma, breast cancer, esophageal cancer, malignant melanoma, etc.	First marketed in 1980	^45,46^
**Vinorelbine** **Ditartrate**	Vinblastine		Non-small cell lung cancer, breast cancer, etc.	First marketed in 1989	^45,46^
**Javlor**	Vinblastine		Urothelial carcinoma, etc.	First marketed in 2009	^47–49^
**DPT**	Podophyllotoxin	–	Non-small cell lung cancer, breast cancer, stomach cancer, etc.	In phase I clinical trials	^61,62^
**Irinotecan**	Camptothecin	Specifically inhibits DNA TopoI, which promotes double-stranded DNA deconvolution, thereby inhibiting DNA synthesis and achieving heterotypic cell growth	Colorectal cancer, lung cancer, breast cancer, pancreatic cancer, etc.	First marketed in 1994	^92,93^
**Topotecan**			Small cell lung cancer, metastatic advanced ovarian cancer that has failed first-line chemotherapy, etc.	First marketed in 1996	^92,93^
**Hydroxycam-ptothecin**			Liver cancer, colorectal cancer, lung cancer, leukemia, etc.	In phase III clinical trials	^94,95^
**Exatecan**			Breast cancer, ovarian cancer, colorectal cancer, etc.	In phase I clinical trials	^94,95^
**Rubitecan**			Most solid tumors, blood cancers, etc.	In phase III clinical trials	^96^
**Flavopiridol**	Rohitukine	Inhibits mitosis by inhibiting cyclin-dependent kinases, thus leading to cell cycle arrest	Esophageal cancer, lung cancer, prostate cancer, etc.	In phase II clinical trials	^167,168^
**UCN-01**	Staurosporine		Lymphoma, etc.	In phase II clinical trials	^169^
**Midostaurin**			Acute myeloid leukemia containing FLT-3 mutation, etc.	First marketed in 2017	^170^
**Roscovitine**	Olomucine		Breast cancer, nasopharyngeal carcinoma, etc.	In phase I clinical trials	^171,172^
**Seliciclib**			Breast cancer, nasopharyngeal carcinoma, etc.	In phase I clinical trials	^171,172^

## Microtubule

**Figure 1:**
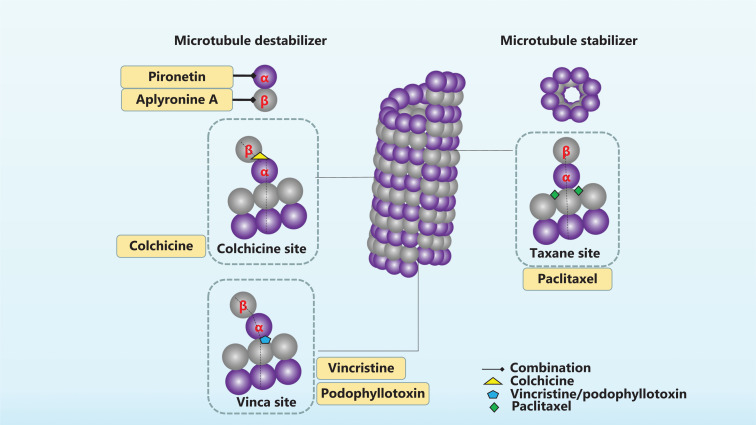
Natural products targeting microtubule proteins. Microtubules are composed of 2 types of microtubule proteins, α- and β-tubules, in a directed arrangement, which further form the spindle. The movement of microtubules is regulated, and inhibition or stabilization of microtubules interferes with mitosis. Paclitaxel is a microtubule stabilizer that binds the Taxane site of β-microtubule proteins. Vincristine, colchicine, podophyllotoxin, and aplyronine A are microtubule destabilizers that bind different sites of β-microtubule proteins. Pironetin acts on α-microtubule proteins and exerts microtubule depolymerizing effects.

Microtubules in eukaryotic cells are polar cytoskeletal structures with crucial roles in cell mitosis, intracellular transport, and many other key cellular events. The cortex of microtubules is often composed of 13 protofilaments, mainly comprising tubulin heterodimers (α- and β-tubulin)^13^. Each heterodimer is arranged in a head-to-tail combination, thus forming directional microtubules and giving rise to the polar character of microtubules. The terminal with β-tubulin is the plus end, and the terminal with α-tubulin is the minus end^6^. Microtubules are hollow tubes that span the entire cell, and facilitate mitosis or intracellular transport^13^. Microtubules compose the spindle and are involved in all processes of mitosis. The kinetochore microtubules of the spindle emanate from the pole at one end and bind the centromeric chromatin at the other end^14^. Chromosomes that undergo replication have two mitoses, and bind two microtubules facing opposite spindle poles, thus achieving biorientation^15^. The aberrant expression of tubulin or microtubule-regulating proteins often occurs during tumor development^7^. For example, the high expression of β-microtubulin, particularly the βIII isoform, promotes tumor differentiation and metastasis. In addition, elevated levels of βIII-microtubulin affect resistance to broad-spectrum drugs and non-microtubule-targeting drugs^16,17^. Natural products targeting microtubule proteins are shown in **Figure 1**.

### Paclitaxel (Taxol)

Paclitaxel is a secondary metabolite isolated from the bark of the Pacific yew *Taxusbrevifolia*. Paclitaxel promotes the assembly of tubulin into microtubules and prevents the depolymerization of microtubules, thus further promoting the formation of stable microtubules from microtubulin, and effectively blocking the cell cycle at G2/M phase, preventing mitosis, and ultimately inhibiting proliferation or promoting apoptosis of cancer cells^18^. Recent research has shown that paclitaxel does not directly inhibit cell mitosis but instead induces the production of multipolar mitotic spindles that mediate unbalanced or mis-segregated chromosome distribution^19,20^. This abnormality leads to the formation of aneuploid progeny, loss of important chromosomes in the tumor cells, and ultimately cell death^20^. This mechanism partly explains the reason for paclitaxel insensitivity. Some clinical studies have shown that only approximately half of patients with breast cancer treated with paclitaxel have tumor shrinkage or disappearance. In patients who do not respond to paclitaxel, no clinically relevant paclitaxel is detected, and the mitosis of tumor cells does not cease^21,22^. In paclitaxel-sensitive patients, multipolar spindles form in tumor cells. When the abnormal multipolar spindle is maintained, the tumor cells die. After restoration of bipolar spindles, cancer cells usually become more resistant to paclitaxel.

Although paclitaxel is a highly successful antitumor drug in clinical practice, its disadvantages, such as poor water solubility, poor targeting, and strong non-selective toxic effects, have greatly hindered the development of new paclitaxel drugs^23^. Taxol, a first-generation paclitaxel preparation, has poor solubility, a short half-life, a long injection time, low bioavailability, and a tendency to cause allergic reactions^24–26^. Several new dosage forms of paclitaxel are available on the market. For example, albumin-bound paclitaxel, whose trade name is Abraxane, requires only half an hour to inject and can be used to treat breast cancer^27,28^. In 2007, Samyang launched Cynviloq, the first paclitaxel micelle formulation marketed worldwide. This dosage form has not only extended the half-life but also improved drug solubility. Paclical, which has a much higher drug loading than Cynviloq, was introduced to treat expanded indications^29^. Paclitaxel liposomes for injection are yet another formulation of paclitaxel that can be used as a first-line chemotherapy for ovarian cancer, or in combination with cisplatin^30,31^. In 2016, the Korean drug regulatory agency launched the world’s first paclitaxel oral solution (Liporaxel) for gastric cancer^32^. Subsequently, Hanmi Pharmaceuticals developed Oraxol, an innovative oral formulation of paclitaxel combined with ansquetta mesylate. Clinical studies have shown that Oraxol locally inhibits the activity of p-GP in the intestinal tract, thus improving drug efficacy while decreasing adverse effects elsewhere in the body^33,34^. After the development of new dosage forms of paclitaxel, paclitaxel derivatives followed. Sanofi developed Taxotere to address the problems of poor solubility and allergic reactions of paclitaxel, but this drug tends to cause permanent hair loss^35,36^. In China, in addition to Taxotere, which has been marketed, Jevtana, Larotaxel, Milataxel, and Ortataxel are under clinical trials.

### Vincristine

Vincristine is a potent agent with well-defined effects on hematologic disorders and several types of solid tumors. As a microtubule-destabilizing drug, vincristine impairs the microtubule network by binding the *Vinca* domain in the β-tubulin subunit, thus inhibiting the polymerization of microtubules and arresting mitosis in phase M^37^. Vincristine forms a wedge at the dimer interface between α- and β-microtubulin while bending the microtubule growth axis^38^. Low concentrations restrain mitosis without depolymerization of the spindle in HeLa cells, whereas high concentrations induce rearrangement of spindle microtubules and centrosome rupture, thus arresting mitosis in M phase^39^. The transcription factor MYC, a key regulator of cellular proliferation, has been reported to be associated with highly aggressive cancers and poor prognosis in patients with cancer^40^. MYC overexpression induces mitotic spindle assembly defects through microtubule nucleation and organization^41^. Phosphorylation of MYC occurs in mitosis, but a fraction of non-phosphorylated MYC binds the microtubules of the mitotic spindle^42,43^. Vincristine has been found to destabilize wild-type MYC during mitosis. Furthermore, a strong inhibitory effect of vincristine has been observed in a population of lymphoma cells with high MYC expression^44^. The above results indicate that vincristine may be a good candidate in the treatment of MYC-driven malignancies.

Vincristines are typical antitumor drugs that target microtubules; the 4 main members are vinblastine, vincristine, vinorelbine, and vindesine^45,46^. Because the bioavailability of these 4 drugs is very low, they are administered mainly intravenously as sulfate and tartaric acid in clinical practice. Vinflunine (Javlor), a semi-synthetic derivative of periwinkle alkaloids, is a novel bifluorinated microtubule inhibitor for the treatment of bladder cancer that has failed to respond to chemotherapy^47–49^.

### Colchicine

Colchicine, another microtubule-targeting agent (MTA), is a tricyclic alkaloid extracted from plants of the genus *Colchicum* (*autumn crocus*). This classical anti-mitotic drug binds soluble tubulin at colchicine site between α- and β-subunits, thus irreversibly forming tubulin-colchicine complexes, which induce many conformational changes in tubulin, disrupt further microtubule polymerization, and finally block mitosis at metaphase and induce cell death^50^. A detailed kinetic analysis of the inhibitory interaction has shown that tubulin-colchicine complexes bind the ends of the microtubules and prevent further addition to microtubules by sterically blocking terminal tubulin dimer growth^50^. The mitotic effect of colchicine mediated cell division is only temporary, and when the colchicine inside cells is metabolized, mitosis continues. In addition, the sensitivity of cells to colchicine varies according to its concentration. At low concentrations, colchicine arrests microtubule growth, whereas at higher concentrations, it promotes microtubule depolymerisation^50^. The severe toxicity of colchicine at high doses in normal tissues limits its clinical use as an anti-tumor agent^51,52^. Because colchicine has high anti-proliferative activity and promising clinical prospects, many colchicine analogues have been synthesized to avoid adverse effects^53^. However, because the effective dose of colchicine is close to the toxic dose, it is not commonly used clinically^52^. The main clinical applications of colchicine are currently the treatment of ventilation and cardiovascular diseases^54^. Because of the high toxicity of colchicine, experimental studies have synthesized derivatives with substituted functional groups on the a-ring (methoxy), b-ring (acetamide), and c-ring (methoxy) for potentiation and detoxification^55^.

### Podophyllotoxin

Podophyllotoxin, an aryltetralin-type lignan isolated from species of *Podophyllum*, is widely used to treat a variety of cancers including liver cancer, lung cancer, and neuroblastoma^56^. Similarly to colchicine, podophyllotoxin binds the colchicine site at the interface of α- and β-tubulin, inhibits the assembly of tubulin into microtubules, blocks the cell cycle in G2/M phase, and finally leads to mitosis arrest^57,58^. However, clinical therapy is severely limited because of systemic toxicity effects. Recently, modification of podophyllotoxin has enabled clinical application; the most clinically used drugs are etoposide and teniposide, which are glycosylated at the C-4 position^59,60^. Because podophyllotoxin is an effective targeted microtubule drug, drug development has continued. Deoxypodophyllotoxin (DPT) injection is currently under development in China in phase I trials. Various *in vitro* experimental studies on DPT have indicated its strong killing effect on a variety of human-derived tumor cells^61^. *In vivo* experiments have shown that DPT is effective against a variety of drug-resistant tumor strains, thus making it a valuable new antitumor drug^62^.

### Aplyronine A (ApA)

ApA, a marine natural product originally isolated from *Aplysiakurodai* sea hares, exerts antitumor activity by inducing interactions between actin and microtubulin^63–65^. Actin is a class of globular multifunctional proteins that form microfilaments and are essential in diverse cellular processes, including movement and contraction. The dynamic balance between microtubules and actin is highly important for the stable morphology and mitosis of mammalian cells^66^. ApA specifically induces microtubule breakdown in the presence of actin. Conformation analysis of ApA on actin has indicated that the C13 methoxy group of ApA not only directly interacts with Glu334 of actin through the C9 hydroxyl group but also interacts with Arg147 through water molecules^66^. One study has indicated that ApA and actin synergistically inhibit microtubule polymerization^67^. ApA binds actin inserts its aliphatic tail into the actin molecule and binds the hydrophobic cleft consisting of actin subdomains 1 and 3, thus creating an actin-ApA complex^68^. The complex may further interact with microtubule protein heterodimers (released from the cytoplasm) and form a ternary complex, which microtubule polymerization^65^.

### Pironetin

Pironetin, a Streptomyces polyketide metabolite, is a microtubule destabilizer that blocks cell mitosis in G2/M phase by inhibiting microtubule protein polymerization^69^. Unlike most MTAs, including paclitaxel, vincristine, colchicine, laulimalide, and medensin, pironetin is the only agent crystallographically characterized to bind solely to α-tubulin. Both α- and β-microtubulin bind GTP, but only β-microtubulin has GTPase activity and can hydrolyze GTP to GDP. Consequently, drugs binding the β-subunit are rapidly resistant. In contrast, the α-subunit has no GTPase activity and is not affected by mutations in β-microtubulin. Drugs targeting α-microtubulin have high clinical value. Pironetin inhibits the binding of radiolabeled vinblastine to tubulin, and has a stronger affinity for tubulin than vinblastine^70^. Researchers have found that pironetin disrupts the longitudinal contacts between microtubulin heterodimers and that the disruption may be the basis for promoting microtubule depolymerization. A re-examination of the molecular structure of pironetin has confirmed that pironetin covalently binds Cys316 of α-microtubulin, thus refuting the previous conclusion that it covalently binds Lys352^70,71^. The residues on both sides of Cys316 on the S8 chain differ from the side chain structures of Cys316 and Leu318, which are necessary for the binding of pironetin^71^. The distortion of the precise position of Glu254 and the resultant lower GTPase activity of β-tubulin may be an additional mechanism through which pironetin destabilizes the assembly of tubulin into microtubules^71^.

MTAs interact with tubulin, thus leading to cell death by altering microtubule dynamics. Natural MTAs, such as paclitaxel and vincristine, have long been used in cancer treatment^72^. We summarized the effects and mechanism of natural products targeting microtubulin, including paclitaxel, vincristine, colchicine, podophyllotoxin, ApA, and pironetin. Paclitaxel is a representative microtubule stabilizer that can result in the formation of aneuploid progeny by inducing multilevel spindle production, thus leading to tumor cell death. Others drugs include microtubule inhibitors, which block microtubule polymerization and disrupt microtubules and consequently mitosis. The special compound is pironetin, a recently discovered inhibitor of α-microtubulin, which disrupts the longitudinal contacts between microtubule protein heterodimers, thereby promoting microtubule depolymerization. Although MTAs are potent anti-tumoral drugs, their resistance and dose-limiting toxicity limit their clinical efficacy. To address the shortcomings of microtubule-targeted drugs, researchers have proposed the concept of dual-target microtubulin inhibitors, in which microtubulin inhibitors are combined with other antitumor drugs to achieve synergistic effects^73,74^. In recent years, to overcome the resistance and toxic effects of microtubulin inhibitors, particularly those developed from natural products, researchers have gradually focused on the development of dual-targeted drugs, such as paclitaxel dual-targeted nano-drugs^75–78^. The development of dual-targeted microtubule protein inhibitors is an area of great promise.

## DNA damage

**Figure 2:**
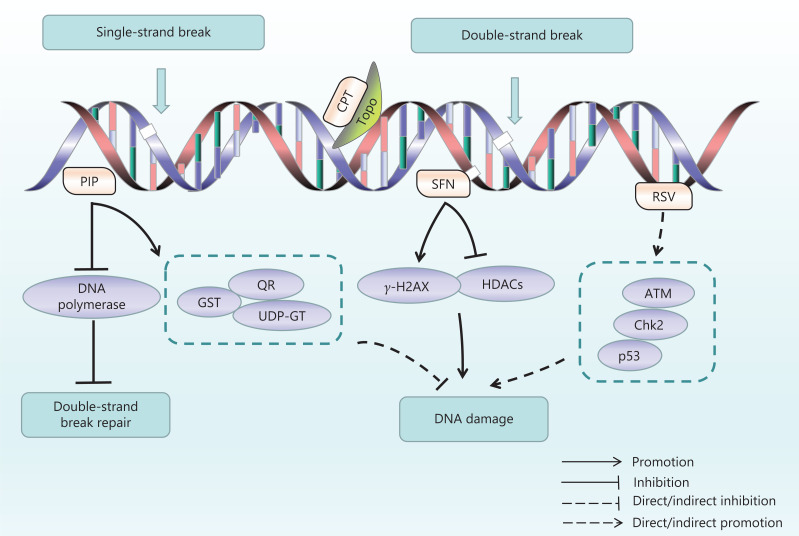
Natural products targeting DNA damage. DNA damage and repair (DDR) are closely associated with mitosis, and many natural products have been found to interfere with DNA damage or DDR. Camptothecin (CPT) directly binds DNA as a topoisomerase inhibitor. Sulforaphane (SFN) inhibits HDAC activity and enhances acetyltransferase expression, thus further causing DNA damage. Resveratrol (RSV) induces ROS accumulation, thereby leading to base double strand breakage. Piperine (PIP) inhibits the expression of DNA polymerase and prevents DDR. PIP also enhances GST, QR, and UDP-GT, and indirectly decreases DNA damage.

DNA damage is a permanent alteration of DNA nucleotide sequences caused by endogenous or exogenous factors, including spontaneous damage to DNA molecules, by-products of cellular metabolism, exogenous free radicals, and topological changes triggered by physical or chemical damage. DNA damage has been recognized as the underlying cause of many diseases, including cancer. Types of DNA damage include nucleotide mutations, substitutions, deletions, insertions, bulky adducts, single-strand breaks, and double-stand breaks (DSBs)^79,80^. DNA damage leads to genomic instability and induces functional mutations in oncogenes or tumor-suppressor genes, which are a causative factor for tumorigenesis^81^. DNA damage evokes responses by multiple repair mechanisms and signaling pathways. The DNA damage response (DDR) involves various intra- and inter-cellular signaling events and enzymatic activities that lead to cell-cycle arrest, regulation of DNA replication, and DNA damage repair^82^. Because genomic instability is a pervasive characteristic of tumor cells, DDR defects can serve as therapeutic targets. DNA damaging chemotherapies have been used in clinical cancer chemotherapy for decades, and include drugs that induce covalent crosslinks between DNA bases, drugs that attach alkyl groups to bases, and drugs that cause single-strand breaks or DSBs by targeting topoisomerase (Topo) I or II enzymes^83^. The natural products targeting DNA damage are shown in **Figure 2**.

### Camptothecin (CPT)

CPT is a pentacyclic alkaloid first isolated from *Camptotheca acuminate*. CPT is a typical TopoI inhibitor that induces S and G2 phase arrest. DNA TopoI resolves the tension caused during the winding and release of the DNA helix by forming a cleavage complex (TopoI covalent complex) that produces a transient DNA single-strand break, thus repairing the double-helix structure of DNA. CPT binds at the interface between TopoI and DNA and inhibits TopoI by binding to the Topo I-DNA complex^84^. TopoI inhibition of CPT is required for binding the TopoI/DNA covalent complex and forming a ternary complex that prevents rejoining of the broken DNA^85,86^. Early studies have demonstrated that CPT acts mainly on the DNA synthesis phase (S phase). CPT also affects pre-G1 phase and late-G2 phase, to a lesser extent than S phase^87^. The targets of CPT vary by concentration: low concentrations of CPT act on G2/M phase, whereas increasing concentrations block more cells in S phase^88^. Treatment with 25-nM CPT delays replication by several hours, whereas 1-μM CPT leads to a persistent replication block and massive cell death. Nanomolar concentrations (low doses) of CPT have been found to stimulate replication-fork slowing and reversal, whereas at high doses (micromolar levels), DSB formation is prevalent^89^. Mitotic arrest deficient 2 (Mad2), a mitotic checkpoint protein, is responsible for verifying the attachment of the mitophagus to the spindle^90^. CPT upregulates Mad2 through the Jun N-terminal kinase (JNK)-mediated SP1 pathway, and promotes microtubule protein polymerization and autophagy, thus leading to mitotic arrest and cell death^91^. Because of the poor solubility and severe toxicity of CPT, more effective CPT derivatives have been developed. Topotecan and irinotecan, water-soluble analogs, have been approved for treating common human cancer types^92,93^. Irinotecan and topotecan are semi-synthetic derivatives of CPT; irinotecan is the first-line drug for advanced colorectal cancer, and topotecan is a drug indicated for the treatment of breast cancer^94,95^. The CPT derivatives exhibit strong antitumor activity and low toxic adverse effects. Other marketed CPT derivatives, including hydroxylCPT and exatecan, have been tested in clinical trials and found to strongly block the effects of DNA synthesis in tumor cells^96,97^. Rubitecan, a second-generation topoisomerase inhibitor, is used to treat blood cancers and a variety of solid tumors^98^.

### Piperine (PIP)

PIP is an acinnamamide alkaloid extracted from *Piper nigrum* and *Piper longum*, a commonly used spice^99^. PIP consists of 2 heterocycles linked by an aliphatic diene system, a structure that enables PIP to modulate multiple targets^100^. PIP exerts anti-tumor effects through a variety of mechanisms, such as inducing G1 cell cycle arrest^101^, inducing abnormal DNA structure, inducing ROS production and consequently disrupting redox homeostasis^102^, inhibiting tumor angiogenesis^103^, and inducing tumor cell autophagy.^104^. PIP directly binds the minor grooves of DNA^105^. Recent research has indicated that PIP shows specificity for G-quadruplex DNA and binds the DNA sequence of the c-myc promoter region (Pu24T). Thus, the abnormal DNA tetraspiral DNA molecule structure causes apoptosis^106^. In addition, in melanoma cells, PIP decreases the expression of DNA polymerase β, an enzyme that plays a role in the repair of DSBs, and prevents the repair of DNA damage. PIP also activates checkpoint kinase 1 (Chk1) and causes G1 phase cell cycle arrest^107^. Furthermore, PIP is well known for its ability to regulate metabolism enzymes, such as cytochrome P450. In an experimental model of benzo(a)pyrene [B(a)p]-induced lung cancer, PIP has been found to enhance the expression of detoxification enzymes, such as glutenzyme detoxification enzyme (GST), anthracene reductase (QR), and UDP-borate transferase (UDP-GT), thus indirectly decreasing DNA damage^108^.

### Sulforaphane (SFN)

SFN, an isothiocyanate found in Brassicaceae vegetables, particularly broccoli, is recognized for its antioxidant capacity and anticancer activity^109^. SFN exhibits cytotoxic activity through several mechanisms including inducing detoxifying enzyme production, inducing ROS production, cell cycle arrest, apoptosis, and epigenetic regulation. Researchers have found that SFN inhibits histone deacetylase (HDAC) activity and enhances acetyltransferase expression, thus further causing DNA damage. Importantly, colon cancer cells are far more susceptible than non-cancer cells to SNF-induced DNA damage^110^. Myzak et al.^111^ first reported that SFN inhibits the activity of HDAC itself without altering the protein level of HDAC and β-catenin. HDAC inhibition has also been reported to disrupt the cell cycle in the G2 phase and interfere with the mitotic spindle checkpoint^112^, effects also caused by SFN. SFN upregulates CDK1 and cyclin B1 in HepG2 cells, which form a complex responsible for the cell cycle transition from G2 to mitosis. The cyclin B1-CDK1 maintenance induced by SFN causes misalignment of chromosomes to the spindle, thus arresting mitosis at prometaphase^113^. SFN-induced ROS generation is another important mechanism causing DNA damage and tumor cell apoptosis. For example, SFN inhibits and disrupts redox homeostasis by depleting glutathione (GSH), thereby inducing ROS production. The increase in expression of γH2AX, a DNA damage marker, after treatment with SFN, indicates the occurrence of DNA damage and a subsequent G0/G1 phase block in esophageal squamous cell carcinoma cells^114^. In fact, SFN exerts dichotomous effects in ROS production. SFN is also an indirect ROS scavenger through enhancing nuclear factor E2-related factor 2 (Nrf2) activity. SFN promotes the nuclear accumulation of Nrf2, which acts as a transcriptional activator via antioxidant response elements^115^.

### Resveratrol (RSV)

RSV, a natural polyphenolic compound found in foods including grapes, berries, peanuts, and red wine, has potent anti-inflammatory, anti-bacterial, and anti-oxidant activities, and also has preventive and therapeutic effects on various cancers, as demonstrated in clinical trials^116,117^. In human head and neck squamous cell carcinoma cells, RSV induces DNA damage, mainly targeting mutant cancer cells without affecting normal cells, a phenomenon that is associated with Smad4^118^. In colon cancer models, RSV single treatment induces DDR correlated with S-phase delay and apoptosis; however, prolonged treatments lead to stable resistance which is triggered by ROS over-production. Research has indicated the importance of RSV-induced ROS production and the indirect DNA-damaging effects, as well as the long-term adaptation of cancer cells toward oxidative stress^119^. In extra nodal natural killer cell/T-cell lymphoma, an aggressive lymphoma with poor prognosis, RSV blocks the cell cycle in S-phase by activating the DDR pathway *via* upregulating the expression of Zta (also referred to as BZLF1, ZEBRA, EB1) of Epstein-Barr virus. The mechanism of DDR activation may involve an ATM/Chk2/p53-dependent pathway^120,121^. Etoposide is an antitumor agent that acts on DNA TopoII, thus causing DNA strand breaks; this chemotherapeutic agent impedes DNA repair^122^. Importantly, RSV synergistically activates the DDR pathway with etoposide, thus suppressing cell proliferation and promoting apoptosis^123^. In addition, studies have shown that RSV acts synergistically with etoposide, a DNA TopoII inhibitor, thereby causing DSBs, activating the DDR, and promoting apoptosis^124^.

DNA damage occurs in gene mutations and cancer development, and subsequently further activates the DDR to repair the damage. If the repair is not completed before mitosis, the damage is passed on to daughter cells and may lead to cellular senescence, apoptosis, and even cancer. Genomic instability is a feature of cancer cells that makes them susceptible to DNA damage. Targeting DNA damage and DDR inhibition holds promise for exploiting this defect in cancer cells, and the development of DNA damage inducers and targeted inhibition of DDR may lead to favorable anti-tumor treatments. The roles of natural products in DNA damage are diverse. Some compounds tend to induce ROS accumulation, thus leading to base double bond breakage; examples include PIP, carotenoids, and RSV. These compounds also indirectly cause DNA damage in tumor cells by regulating other proteins; their effects include the inhibitory effect of PIP on DNA polymerase β and the down-regulation of deacetylase by carotenoids. CPT plays a crucial role in the induction of DNA damage *via* directly binding DNA as a typical topoisomerase inhibitor. These natural products indirectly affect the mitosis of tumor cells by promoting DNA damage, thus leading to cell death. However, the specific mechanisms of most natural products in DNA damage remain unclear, and the identification of specific molecular targets is lacking.

## Mitosis-associated kinases

In addition to targeting microtubules and targeting DNA damage, as described above, another highly important strategy is targeting mitosis-associated kinases. Mitosis cannot proceed properly without many protein phosphorylation events, which are regulated by protein kinases^125,126^. The protein kinases described in this review are CDK and AURK, which are frequently overexpressed in cancer cells and result in uncontrolled proliferation. In addition, recent studies have found that many natural products directly control the mitotic process of cancer cells by regulating these 2 kinases.

### CDKs

CDKs drive the cell cycle primarily through chemotaxis on serine/threonine proteins, and act synergistically with cyclins, important factors in the regulation of the cell cycle. The cell division cycle is a strictly controlled process that leads to the duplication of genomic DNA and the reproduction of 2 daughter cells. The cell cycle is divided into 4 main phases: (1) G1 (gap 1) phase, known as prophase synthesis, which involves the synthesis of RNA and ribosomes in preparation for S phase; (2) S (synthesis) phase, involving synthesis of DNA and histones; (3) G2 (gap 2) phase, involving late DNA synthesis and preparation for mitosis; and (4) M (mitosis) phase, in which the cell divides the duplicated DNA and cellular components equally between daughter cells^127^. The cell cycle involves numerous CDKs, which form complexes with cyclin proteins and subsequently regulate cell mitosis^128^. Specific CDK–cyclin complexes drive various events in a sequential and orderly manner. During G1 phase, cyclin D senses mitogenic signals, its expression increases and it binds CDK4/6; the complex formed subsequently phosphorylates and inactivates the retinoblastoma (RB) family proteins. The partial inactivation of RB proteins allows for the expression of E-type cyclins (E1 and E2), which bind CDK2. CDK2–cyclin E complexes further phosphorylate and completely inactivate RBs, thereby releasing more E2F (a transcription factor) and activating the G1/S checkpoint^129,130^. CDK2 is subsequently activated by cyclin A, then drives the transition from S to G2 phase. In S phase, the accumulatied CDK2-cyclin A complex phosphorylates tricarboxylic acid (TCA) cyclase, thus enhancing glycolysis and providing energy for DNA replication^129,131^. Finally, CDK1 is activated by cyclin A and consequently promotes mitotic entry. After nuclear envelope rupture, cyclin A is degraded, thereby promoting the formation of the CDK1-cyclin B complex, which drives the cell cycle to mitosis^128,129^.

**Figure 3:**
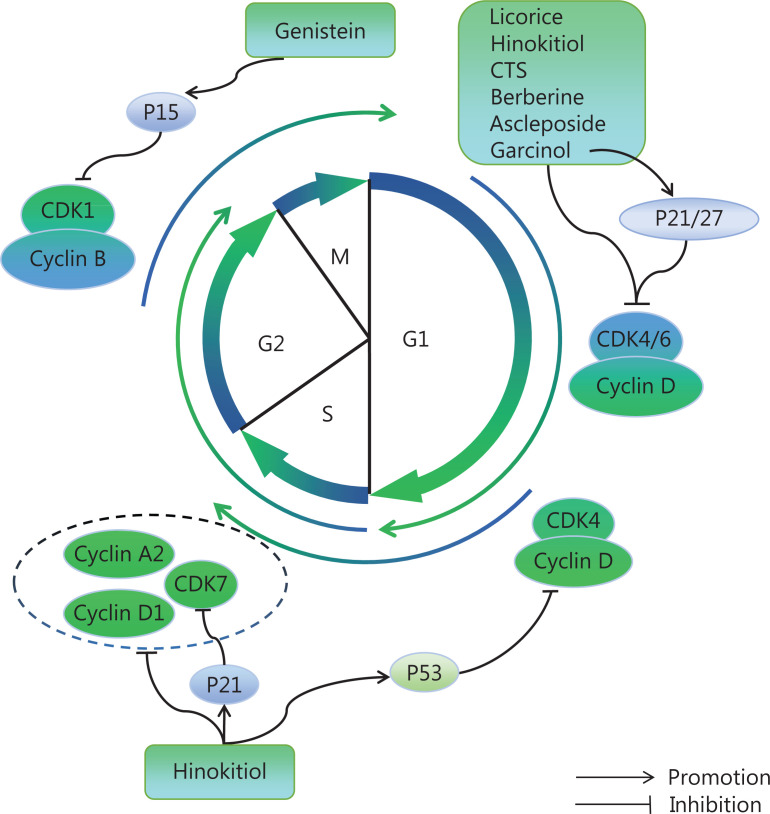
Natural products regulating the cell cycle. An organized cell cycle is the basis for cell division. Many natural products act as cell cycle blockers *via* targeting cyclin proteins and CDKs. Licorice, hinokitiol, cryptotanshinone (CTS), berberine, ascleposide, and garcinol block the cell cycle in G0/G1 phase. Genistein blocks the cell cycle in G2/M phase and promotes apoptosis. Hinokitiol also decreases CDK7, cyclin D1, and cyclin A2, thereby blocking S phase.

The cell cycle is commonly dysregulated in neoplasia, and the specific transcription of cell cycle proteins is usually aberrantly activated; cells subsequently undergo continued proliferation or unscheduled re-entry into the cell cycle^132^. In most tumors, CCND family members (encoding cyclin D) and CDK4/6 are overexpressed, thus eliminating RB-mediated cell cycle inhibition and leading to uncontrolled cell proliferation^133,134^. In addition, tumor development is usually accompanied by the up-regulation of cyclin E, which has a high affinity toward CDK2. The role of cyclin E in tumor development has been fully confirmed, whereas the role of CDK2 has not yet been elucidated^135^. Many studies have indicated that CDK2 inhibition retards malignancies, such as melanoma, ovarian cancer, and diffuse large B-cell lymphoma. The expression of cell cycle-related proteins is altered to some extent in most cancer cells, such as cyclin A, cell division cycle gene7 (CDC7), cyclin B-CDK1, and cyclin H-CDK7^136^. Natural products targeting CDKs are shown in **Figure 3**.

#### Licorice

*Glycyrrhiza uralensis* Fisch (licorice) is a versatile herb whose active coupounds are mainly flavonoids and triterpene saponins. Licorice has anti-tumor effects through modulating the cell cycle or the immune microenvironment. A previous report has indicated that licorice inhits the proliferation of H1975 non-small cell lung cancer (NSCLC) cells and MCF-7 breast cancer cells in a dose-dependent manner, potentially through the down-regulation of cyclin D1-CDK4 complex expression, and blocks tumor cells in G0/G1 phase. The down-regulation of cyclin D1-CDK4 increases antigen presentation and induces infiltration of CD8^+^ T cells into tumors, thereby exerting anti-tumor effects^137^. Licochalcone A, a flavonoid isolated from licorice with anti-tumor properties, has been reported to inhibit the proliferation of HepG2 cells by terminating the cell cycle at the G2/M transition, as characterized by the decreased expression of cyclin B1 and CDK1^138^.

#### Hinokitiol

Hinokitiol, a natural monosteroidal compound with chro-phenolic ketone structure extracted from the trunks of *Chamaecyparisobtusa*, is often used as a potent fungicide. In recent years, hinokitiol has been found to have notable anti-tumor activity, including inhibiting tumor cell migration, promoting cell apoptosis, and inducing autophagy, thus highlighting its great potential for treating tumors. Hinokitiol treatment of endometrial carcinoid cells increases p53, decreases cyclin D1 and CDK4, and blocks cell growth in G0/G1 phase^139^. In human hepatocellular carcinoma cells, hinobinol mainly causes a decrease in CDK7, cyclin D1, and cyclin A2, thus blocking the S phase^140^.

#### Cryptotanshinone (CTS)

Cryptotanshinone (CTS), a lipophilic compound extracted from *Salvia miltiorrhiza* Bunge, shows anti-tumor activity. *In vitro* experiments have indicated that CTS inhibits the viability of human NSCLC cells (A549 and H460), partly *via* down-regulating cyclin A/D and CDK2/4, thus blocking the cell cycle in G0/G1 phase^141^. Furthermore, CTS down-regulates IAP family proteins and apoptosis inhibitors, and promotes apoptosis, thus indicating a close relationship between CTS-induced cell cycle arrest and apoptosis.

#### Genistein

Genistein, an isoflavone extracted from *Genista tinctoria* Linn, exerts anti-tumor effects through apoptosis, cell cycle arrest, and inhibition of angiogenesis^142^. Genistein promotes apoptosis by inhibiting the activation of the PI3K/Akt signaling pathway. Cyclin A/B downregulation and binding CDK2 and CDC2 then block the cell cycle in G2/M phase and promote apoptosis^143^. In addition, in other tumor types, genistein indirectly plays a different role as a CDK inhibitor. In uterine smooth muscle tumors, genistein promotes expression of p15, a CDK inhibitor^144^. In colorectal cancer, the action of genistein is associated with the elimination of CDK2 phosphorylation^145^. In ovarian cancer, genistein mainly inhibits cyclin D1 expression but enhances p21 expression^146^.

#### Berberine

Berberine is a natural quaternary alkaloid isolated from *Coptis chinensis* Franch, with anti-inflammatory, anti-oxidant, and anti-tumor effects^147^. *In vitro*, berberine has been found to significantly decrease the expression of cyclin D1, D2, and E in DU145 human prostate cancer cells, with the most pronounced effect on cyclin D1. The down-regulation of CDK2, CDK4, and particularly CDK6 has also been observed^148^. The inhibition of cyclin D and CDKs by berberine indicates inhibition of of cyclin D-CDK6 complex formation, thus blocking the cell cycle in G1 phase.

#### Ascleposide

Ascleposide, a natural cardiac steroid lactone isolated from *Saussurea lappa*, has been found to inhibit DU145 prostate cancer cell growth^149^. Ascleposide downregulates the expression of cyclin D1, cyclin A, and CDK4 in a dose-dependent manner, and also downregulaets c-Myc (an early identified oncogenes) in human prostate cancer cells^150^. C-myc indirectly regulates the cell cycle through the dissociation of the CDK repressor protein p21 from the CDK4/6-cyclin D complex^151^. Therefore, ascleposide exerts indirect and direct effects on cell cycle regulation, and decreases the G1 distribution of cells while increasing the proportion of cells in G2/M and sub-G1 phase^150^. The p21 and p27 Cip/Kip proteins are crucial CDK-interacting protein/kinase inhibitory proteins in mitotis^152^. Interestingly, ascleposide downregulates these Cip/Kip proteins rather than upregulating them^150^, possibly because p21 and p27 resist the apoptotic response in cancer cells^153,154^, thus explaining the ascleposide-induced p21 and p27 downregulation, and anticancer activity in CRPC cells^150^.

#### Others

Garcinol, a polyisoprene benzophenone extracted from the fruit of Garcinia indica, exerts anti-inflammatory and anti-cancer activities. Garcinol induces decreases in cyclin D1, D3, CDK2, and CDK4, and increases in cyclin E and CDK6 in human NSCLC cells (H1299)^155^. In addition, Garcinol upregulates the CDK repressor proteins p21^Waf1/cip1^ and p27^Kip1^, thus inhibiting cyclin-CDK complex assembly. P21^Waf1/cip1^ knockdown results in a complete cell cycle block in G1 phase, thus indicating that the garcinol-induced G1 block in H1299 cells is achieved by upregulation of p21^Waf1/cip1^ protein expression. However, the effect is unclear in H460 cells^155^. Therefore, the mechanism underlying garcinol-induced cell cycle arrest requires further investigation. Similar G1 arrest by 13-O-acetylsolstitialin A and Tetrandrine has been observed, both of which act on the cyclin D1-CDK4 complex: 13-O-acetylsolstitialin A decreases the expression of cyclin D1 and CDK4^156^, whereas Tetrandrine binds the cyclin D1-CDK4 complex and forms hydrogen bonds^157^.

Many natural products also induce G2/M arrest, but their related molecular mechanisms are not well studied. Examples include *Polygonatum cyrtonema* Hua^158^, *Paris chinensis* dioscin^159^, Lycorisaurea agglutinin^160^, *Strychni Semen* (ESS)^161^, and 6-shogaol^162^. Some cause clear S phase arrest by downregualting cyclin A and CDK2 (Juglanthraquinone C^163^ and tea polyphenols^164^) or activating CDK repressor proteins (*Litchi chinensis* Sonn^165–167^ and Gynostemma L^168^).

Normal progression of the cell cycle is necessary to ensure that mitosis occurs without error. The disruption of cell cycle control is a common feature in human cancers^169^. Many natural products can block the cell cycle, including licorice, Hinokitiol, and Cryptotanshinone. However, most studies have been limited to examining their effects on cyclins and kinases, whereas in-depth molecular studies are lacking. Moreover, the effects of most compounds have not been confirmed *in vivo*.

In recent years, clinical development of natural product-derived CDK inhibitors has become more difficult, although increasing natural products have been discovered. Flavopiridol, the first inhibitor of CDKs to enter clinical trials worldwide, is a semi-synthetic flavonoid derivative^170^. However, in phase II clinical trials, researchers have found that it did not show single drug activity in a variety of cancers but showed very significant dose-limiting toxicity^171^. Consequently, Flavopiridol has not been marketed to date. UCN-01 is a derivative of staurosporine that is currently in phase II clinical trials. It binds protein kinase competitively with ATP and has a good selective effect on kinases^172^. Modification of the structure of astrosporin has led to the discovery of a derivative with multi-target kinase inhibitory activity, midostaurin, which was successfully marketed in 2017^173^. Roscovitine and seliciclib are tautomers derived from the natural product olomoucine. Seliciclib has stronger CDK inhibitory activity than Roscovitine, and it blocks RNA polymerase II phosphorylation by inhibiting CDK7 and CDK9 activity^174,175^. Currently, seliciclib has entered phase I and II clinical trials as a drug candidate.

### Aurora kinase

The AURK family is an important class of serine/threonine kinases responsible for the regulation of cell mitosis. Its 3 members are known as Aurora-A (AURKA), Aurora-B (AURKB), and Aurora-C (AURKC) in mammalian cells. AURKA is responsible for regulating mitotic spindle assembly and centrosome segregation, as well as facilitating the transition of cells from G2 to M phase. AURKB is the catalytic component of the chromosome pericentromere complex, which regulates chromatin protein modifications, and promotes chromosome segregation and cytoplasmic division. AURKC is expressed in testis cells and is thought to be important for chromosome segregation during meiosis. AURKA and AURKB play key roles in mitosis.

**Figure 4:**
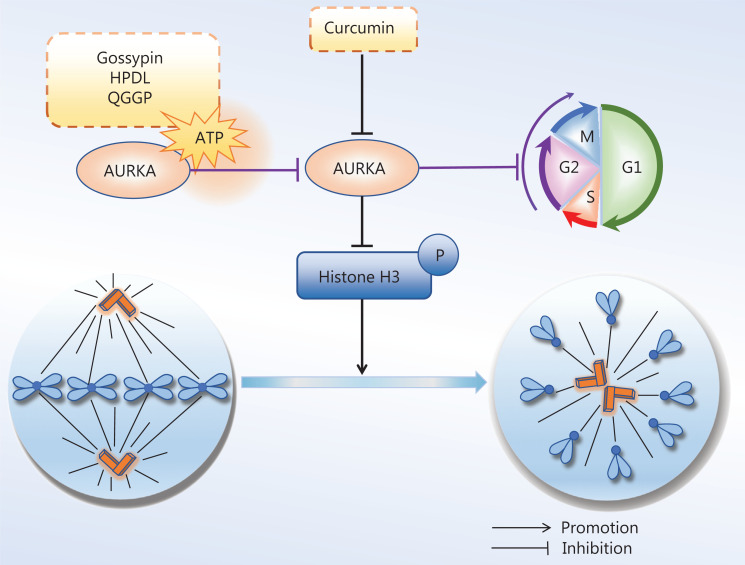
Natural products for targeting AURKs to inhibit mitosis. Aurora kinase (AURKs) are involved in mitotic processes, and down-regulation of AURKs in cancer cells inhibits mitosis. Curcumin inhibits AURKA and changes the bipolar spindle to unipolar, thus inhibiting the continuation of mitosis. Gossypin, as well as 2 active components of eriocaulon sieboldianum (HPDL and QGGP), binds the ATP pocket of AURKA, thereby depleting AURKA.

These kinases are associated with tumorigenesis and are often overexpressed in tumor cells. For example, AURKA is usually highly expressed and actively repairs damaged DNA, thus prompting a return to mitosis. AURKA overexpression constitutively activates CDK1 and abolishes the G2/M DNA damage checkpoint in cancer cells^176^. In addition, AURKA promotes the proliferation and migration of cancer cells during mitotic interphase. AURKB is also a mitotic driver, is overexpressed in a variety of tumor tissues, and promotes tumor invasion. Although AURKC executes unique roles in meiosis, those roles are not discussed here. Given that AURK overexpression is associated with tumorigenesis, these kinases have been potential targets for cancer therapy, and several AURK inhibitors have been developed. In recent years, an increasing number of natural products have shown similar anti-tumor effects to AURK inhibitors. Natural products targeting AURKs are shown in **Figure 4**.

#### Curcumin

Curcumin, a hydrophobic polyphenol extracted from rhizome herbs such as Zingiberaceae Martinov and AraceaeJuss, has a broad spectrum of biological and pharmacological activities, including anti-inflammatory and anti-tumor effects. Curcumin has been found to decrease the AURKA protein and kinase activity in human breast chemoresistant nonmetastatic MCF-7 and highly metastatic cancer MDA-MB-231 cells. The effects of curcumin are similar to those of AURKA siRNA treatment, including monopolar spindle formation, S and G2/M arrest, and diminished cell division^177^. In human bladder cancer cells, curcumin down-regulates the promoter activity of AURKA, thus inhibiting AURKA at the transcriptional level. In addition, curcumin inhibits the phosphorylation of AURKA and its downstream target histone H3 along with the formation of a monopolar spindle. Eventually, G2/M phase arrest occurs, thus decreasing cell division^178^. In summary, the mitotic inhibitory effects of curcumin on cancer cells may be closely associated with the down-regulation of AURKA. In addition to targeting AURKA, curcumin inhibits mitosis by promoting DNA damage, including DNA double-strand breaks^179^, and activation of DDR and pathways^180^. Thus, curcumin is an anti-tumor natural product worthy of in-depth study.

#### Tanshinone I

Danshen (*Salvia miltiorrhiza* Bunge) has been widely used in China for centuries to treat coronary heart diseases. Tanshinones such as cryptotanshinone (CT), tanshinone IIA (T2A), and tanshinone I (T1), are diterpene compounds in Danshen that have been shown to possess anti-tumor activity. Among the studied tanshinones, T1 has strong anti-tumor effects partially attributed to AURKA. In lung cancer, T1 significantly inhibits cell proliferation and tumor growth, but this effect is dramatically eliminated by AURKA knockdown^181^. T1 has shown similar effects in prostate cancer^182^ and breast cancer^183^. In particular, in breast cancer cells (MDA-MB231), only T1, but not other tanshinones, shows a potent inhibitory effect on cell growth; therefore, T1 causes cell cycle arrest in both estrogen-dependent and estrogen-independent cells. T1 regulates the expression of cyclin D, CDK4 and cyclin B, and inhibits the expression of AURKA. Tanshinones appear to have few adverse effects on normal mammary epithelial cells^183^. In summary, AURKA is an important target for tanshinones.

#### Gossypin

Gossypin, a flavone originally isolated from *Hibiscus vitifolius*, has many pharmacological activities, including anti-angiogenesis, anti-inflammation, and anti-tumor functions^184^. Computational docking results have indicated that gossypin directly binds the ATP pocket of AURKA, thus exerting inhibitory effects on AURKA. In human gastric cancer cells, Gossypin inhibits AURKA and induces a G2/M phase block, thereby leading to apoptosis^185^. A similar binding ATP pocket of AURKA has been found in 2 active components of Eriocaulon sieboldianum (HPDL and QGGP) leading to G2/M phase arrest in HepG2 cells^186^.

#### Others

P3, a high molecular weight compound isolated from a marine sponge called *Crambetailliezi*, selectively inhibits AURKA and AURKB, and decreases the mitotic index and apoptotic phenotype of U-2 OS sarcoma cells^187^.

Some TCM formulas or single herbs have also shown inhibitory effects on AURKA, although the active compounds remains unclear. San Huang Decoction (SHD), a Chinese herbal formula made from 5 Chinese herbs, has been widely used to treat metabolic syndrome for centuries and has been used for clinical treatment of patients with breast cancer^188^. SHD has been found to inhibit the proliferaiton of MCF-7 and MDA-MB-231 human breast cancer cells, an effect associated with the down-regulation of AURKA^189^. In addition, by targeting AURKA, SHD may inhibit tumor angiogenesis in breast cancer^190^. *Hedyotis diffusa* Willd. (H) and *Scutellaria barbata* D.Don (S) are 2 ancient anti-cancer Chinese herbs that are used in combined “HS” treatment in prostate cancer^190^. HS inhibits the G2 to M phase transformation of prostate cancer cells by decreasing the protein and mRNA levels of AURKA, CDK1, PLK1, and Cyclin B1^190^. The active components and molecular mechanisms of these herbs require more extensive research.

AURKs are crucial in the spindle assembly checkpoint, alignment of metaphase chromosomes, and chromosomal biorientation. AURKA and AURKB play key roles in mitosis, and some natual products targeting AURKA and AURKB exert anti-tumor effects *in vitro*. Curcumin, tanshinone I, and Gossypin act on AURKA, and P3 acts on AURKA and AURKB *in vitro*. Some TCM formulas, such as SHD, show similar effects on AURKA. However, the mechanism and efficacy *in vivo* require further in-depth study.

Althrough natural products with AURKA inhibitory effects are gradually being discovered, no AURKA inhibitors have been marketed to date or entered clinical trials, owing to the multi-target effects, weak selectivity, and strong toxic adverse effects of natural products. Currently, highly selective AURKA inhibitors have become a focus of new drug development and clinical research.

### Other mitosis-related kinases

Polo-like kinase 1 (PLK1), a new target for mitosis discovered in recent years, belongs to a group of serine/threonine kinases widely found in eukaryotic cells, and is mainly regulated by G1-phase transcriptional repressors and G2-phase transcriptional activators^191^. Recent studies have revealed that the overexpression of PLK1 not only promotes multiple defects in mitosis but also leads to the accumulation of chromosomal instability, thus favoring aneuploidy and tumor formation^192^. PLK1 is a promising target for inhibiting mitosis in cancer cells. *Scutellaria baicalensis* Georgi has strong anti-PLK1 activity, and its main active components, baicalin and baicalin, covalently bind PLK1 *via* Cys133, thus providing a basis for further development of drugs targeting PLK1^193^. *In vitro* experiments with Glucocappasalin have demonstrated its ability to inhibit PLK1 expression in HeLa cells and to induce cell cycle G2/M phase arrest^194^. In addition, Jerantinine B^195^, caffeic acid phenethyl ester,^196^ and catechins^197^ have anti-PLK1 effects in various types of cancer cells.

Monopolar spindle1 (Mps1) is a serine/threonine kinase that ensures proper orientation of sister chromatids on the mitotic spindle, mainly through the spindle assembly checkpoint^198,199^. Treatment of mitotic colorectal cancer cell lines with Mps1 inhibitors results in cell death^200^. T-LAK cell-originated protein kinase (TOPK) is a serine/threonine kinase in the mitogen-activated protein kinase kinase (MAPKK) family that plays a role in cell cycle and mitotic progression^201^. Although little research has been performed on the natural products that regulate Mps1 and TOPK, these kinases are of great interest as promising targets for mitosis, and the discovery of regulatory natural products is believed to provide a reliable basis for clinical trials.

## Discussion

Mitosis is an essential and conserved process in the life cycle of mammalian cells, and is uncontrolled in cancer cells. Thus, interfering with mitosis was an early concept for cancer therapy that has been demonstrated to be successful in clinical settings. In recent years, natural products have attracted increasing attention in cancer therapy, owing to their structural and target diversity. This review summarized the clinical uses of experimental anti-tumor natural products targeting multiple elements during mitosis, including microtubules, the DDR, CDKs, and AURKs. Although many anti-tumor natural products have achieved good clinical effects—including paclitaxel, CPTs, and vincristine—drug resistance, poor targeting, and poor druggability are important factors limiting the clinical application or drug development.

Drug resistance is a major problem in clinical settings. A typical representative of resistance is paclitaxel, a drug that issensitive to βIII-microtubulin^16^. Studies have confirmed that the high expression of βIII in tumor cells is an important reason for paclitaxel resistance, in cancers including lung cancer, ovarian cancer, breast cancer, and gastric cancer. Overexpression of tubulin-βIII decreases the ability of paclitaxel to suppress microtubule dynamics, thus resulting in resistance^202^. Another study on the cytotoxicity of HT29-D4 paclitaxel human colorectal cancer cells has demonstrated that undifferentiated and differentiated cancer cells differ in their sensitivity to paclitaxel, owing to altered βIII-microtubule protein expression. Furthermore, the selective overexpression of tubulin αII and tubulin βI and βIV accounts for the resistance of vincristine^203^, in accordance with the conclusion that differential overexpression of the tubulin isotype is a cause of drug resistance. Resistance to CPTs, DNA TopoI inhibitors, remains not completely clear. The structural alteration of TopoI and cellular response alterations in CPT-DNA-ternary complex formation are the 2 factors in drug resistence^204^. A growing body of research is focusing on the therapeutic targets to ameliorate drug resistance. For example, in the treatment of triple-negative breast cancer, paclitaxel resistance can be reversed by decreasing LIN9 expression, thus suggesting that paclitaxel resistance in triple-negative breast cancer can be reversed by combining bromo- and extra-terminal domain inhibitors with paclitaxel. In addition, compared with β-tubulin-targeted MTA, α-targeted natural products (such as Pironetin) have better selectivity and therefore are in urgent need of development.

Another crucial factor limiting clinical application or use in other therapeutic areas is toxicity toward normal cells. A major problem in using MTA in cancer treatment is its high systemic toxicity, including bone marrow suppression, gastrointestinal bleeding, hair loss, and tingling of the hands and feet. For example, the major toxicities caused by paclitaxel are neutropenia and peripheral neuropathies. Colchicine has been used to treat gout for many years, but its high toxicity at anti-tumor doses, possibly because of its quasi-irreversible binding tubulin, has limited its anticancer therapy development. Because of colchicine’s severe toxicity, many colchicine analogues have been developed to decrease toxicity and maintain efficacy. Studies have shown that modifying colchicine substituents alters binding affinity, and thus the inhibition of tubulin polymerization and cytotoxicity. A simple analog “AC,” retaining only the A and C-ring of colchicine, binds tubulin instantaneously and reversibly, owing to the low activation energy of colchicine–tubulin binding^50^. Different functional groups, such as amides and thioamides, have been introduced in colchicine. An A-ring derivative, 4-halo-(-)-colchicine shows strong activity against human cancer cells, including lung adenocarcinoma A549, colon adenocarcinoma HT29, and HCT116, with the high selectivity and the low toxicity^205^.

The aqueous insolubility or poor pharmacokinetic properties of some natural products have limited their clinical efficacy. Structural modification is one method to increase solubility. For example, the low aqueous solubility and bioavailability of CPT has been largely addressed with chemically modified topotecan and irinocand. In addition to structural modification of natural products, novel delivery systems, such as nanoparticles, provide another way to address current challenges. Various nanoparticles have been developed for delivery of anti-tumor active compounds^206^. Researchers have developed PCL-TPGS (caprolactone-Tocopherol Polyethylene Glycol Succ)-loaded paclitaxel, and the area under the curve of paclitaxel increased about 2.7-fold, the t_1/2_ increased, and the clearance rate decreased compared to free paclitaxel^207^. Similarly, compared with free curcumin, phospholipid-, chitosan- or lipid nanocapsule-loaded curcumin show greater areas under the curve and Cmax, and are more efficiently delivered into tumor cells^208–210^. Moreover, beyond improving the solubility and pharmacokinetic properties, some nanoparticles further enhance the stability and selectivity of drugs, thereby increasing the efficacy or decreasing the toxicity.

In the face of problems associated with the development of clinical drugs of natural product origin, researchers have modified dosage forms and structures to increase efficacy and decrease toxicity. Regarding clinical drugs targeting microtubules, the classical example is paclitaxel, although its insolubility and drug resistance are a major challenge. Several new dosage forms and new structures have been developed to solve the problem during a 50-year-long research process. The emergence of upgraded dosage forms, such as albumin paclitaxel, paclitaxel liposomes, paclitaxel micelles, paclitaxel nanoformulations, and paclitaxel oral formulations, has expanded the indications for paclitaxel drugs. Beyond dosage form modification, many structural modifications to paclitaxel have been developed, such as cabazitaxel, docetaxel, milataxel, ortataxel, salutaxel, simotaxel, and paclitaxel ceribate. The development path of paclitaxel provides a good example for other natural products to solve the clinical development problems of natural products through dosage form modification and structural modification. Beyond the problems of high toxicity, low bioavailability, poor solubility, and high drug resistance of some natural products, the clinical drug development of natural products also faces the problem of unclear multiple targets. Although the multi-target characteristics of natural products can decrease drug resistance, they complicate mechanistic studies. Currently, many new techniques for target prediction are available, such as reverse molecular docking and 3D pharmacophore modeling techniques^211,212^. Wei et al.^213^ have used the parameters of protein-ligand conformation interaction patterns as important indicators for target prediction and screening; this new strategy for target prediction effectively improved the accuracy of small molecule target identification.

In summary, many anti-tumor natural products have been introduced to date, and their anti-tumor activity and molecular targets are receiving increasing attention. Here, we focused on the inhibition of mitosis in tumor cells. Paclitaxel, CPT, and vincristine are typical examples of natural anti-tumor drugs, which have favorable anti-mitosis effects. As the mechanisms of these drugs have been explored, new discoveries have provided clinical advantages. The potential of natural products in anti-tumor drug development is enormous. Simultaneously, natural products have disadvantages such as toxicity and low efficacy, which must be improved in the future. Currently, drug development is increasingly becoming inclined toward structural modification and dosage modification of natural products with significant antitumor effects, and many new drug delivery systems have been developed to expand the drug delivery capacity and improve drug targeting. This review is intended to provide ideas for researchers to discover or develop more potent and selective anti-cancer agents.
